# Supplementary material for: Assessment of peak bone mineral density and its associated factors in Vietnamese adults: A cross-sectional study
Source: PLoS One. 2026 Apr 10;21(4):e0346170. doi: 10.1371/journal.pone.0346170 (PMC13068321; doi:10.1371/journal.pone.0346170)
Supplement: S1 Table — (DOCX) [file pone.0346170.s001.docx]

**S1 Table. Characteristics of study subjects (Male, n=410)**

|  | **<20** | **20-29** | **30-39** | **40-49** | **50-59** | **60-65** | **>65** | **p-value** |
| --- | --- | --- | --- | --- | --- | --- | --- | --- |
|  | **n=39** | **n=91** | **n=26** | **n=37** | **n=52** | **n=29** | **n=136** |  |
| Age | 18.1 (1.6) | 22.7 (1.5) | 35.8 (2.6) | 45.2 (3.0) | 54.6 (2.6) | 62.5 (1.7) | 77.2 (8.3) | <0.001 |
| Weight | 58.7 (12.5) | 63.4 (11.5) | 63.1 (10.4) | 58.6 (9.9) | 60.0 (10.4) | 57.7 (9.5) | 53.9 (9.2) | <0.001 |
| Height | 167.6 (9.0) | 168.8 (6.5) | 164.9 (7.6) | 163.7 (7.8) | 162.6 (5.5) | 161.4 (5.2) | 160.1 (7.4) | <0.001 |
| BMI | 20.81 (3.51) | 22.17 (3.47) | 23.12 (2.71) | 21.89 (3.53) | 22.60 (3.44) | 22.13 (3.53) | 20.99 (3.10) | 0.003 |
| BMD LS *(missing =2)* | 0.980 (0.140) | 0.976 (0.122) | 0.963 (0.151) | 0.966 (0.146) | 0.898 (0.160) | 0.922 (0.218) | 0.888 (0.204) | 0.001 |
| BMD TH *(missing =4)* | 1.061 (0.169) | 1.038 (0.156) | 0.994 (0.165) | 1.052 (0.138) | 0.973 (0.135) | 0.925 (0.148) | 0.885 (0.169) | <0.001 |
| BMD FN *(missing =3)* | 0.996 (0.170) | 0.957 (0.203) | 0.873 (0.127) | 0.891 (0.145) | 0.823 (0.117) | 0.789 (0.164) | 0.761 (0.173) | <0.001 |
