# Supplementary material for: Assessment of peak bone mineral density and its associated factors in Vietnamese adults: A cross-sectional study
Source: PLoS One. 2026 Apr 10;21(4):e0346170. doi: 10.1371/journal.pone.0346170 (PMC13068321; doi:10.1371/journal.pone.0346170)
Supplement: S2 Table — (DOCX) [file pone.0346170.s002.docx]

**S2 Table. Characteristics of study subjects (Female, n=968)**

|  | **<20** | **20-29** | **30-39** | **40-49** | **50-59** | **60-65** | **>65** | **p-value** |
| --- | --- | --- | --- | --- | --- | --- | --- | --- |
|  | **n=17** | **n=64** | **n=28** | **n=77** | **n=185** | **n=166** | **n=431** |  |
| Age | 18.1 (1.6) | 22.7 (1.8) | 37.0 (2.6) | 45.3 (2.7) | 54.5 (2.7) | 62.3 (1.8) | 76.7 (7.8) | <0.001 |
| Weight | 51.4 (7.7) | 50.3 (7.8) | 50.1 (7.5) | 53.2 (6.7) | 54.9 (7.7) | 52.5 (7.8) | 49.8 (8.9) | <0.001 |
| Height | 155.6 (6.0) | 157.4 (5.9) | 155.2 (3.7) | 153.4 (5.3) | 153.6 (6.4) | 151.0 (6.6) | 148.3 (6.6) | <0.001 |
| BMI | 21.14 (2.43) | 20.25 (2.58) | 20.85 (3.24) | 22.60 (2.51) | 23.27 (2.96) | 23.05 (3.17) | 22.64 (3.71) | <0.001 |
| BMD LS  *(missing =10)* | 0.982 (0.173) | 0.967 (0.100) | 0.979 (0.142) | 0.958 (0.126) | 0.855 (0.177) | 0.745 (0.140) | 0.696 (0.165) | <0.001 |
| BMD TH  *(missing =21)* | 0.939 (0.283) | 0.972 (0.109) | 0.990 (0.129) | 1.014 (0.132) | 0.926 (0.146) | 0.851 (0.124) | 0.743 (0.154) | <0.001 |
| BMD FN  *(missing =19)* | 0.934 (0.197) | 0.871 (0.107) | 0.892 (0.109) | 0.893 (0.141) | 0.781 (0.132) | 0.714 (0.105) | 0.630 (0.130) | <0.001 |
