# Supplementary material for: Assessment of peak bone mineral density and its associated factors in Vietnamese adults: A cross-sectional study
Source: PLoS One. 2026 Apr 10;21(4):e0346170. doi: 10.1371/journal.pone.0346170 (PMC13068321; doi:10.1371/journal.pone.0346170)
Supplement: S3 Table — (DOCX) [file pone.0346170.s003.docx]

**S3 Table. Correlation analysis of varibles stratified by gender.**

|  | Lumbar Spine (LS) | Total Hip  (TH) | Femoral Neck (FN) | Age | Weight | Height |
| --- | --- | --- | --- | --- | --- | --- |
| **Male** |  |  |  |  |  |  |
| Lumbar Spine (LS) | 1.000 |  |  |  |  |  |
| Total Hip  (TH) | 0.675* | 1.000 |  |  |  |  |
| Femoral Neck (FN) | 0.583* | 0.822* | 1.000 |  |  |  |
| Age | -0.222* | -0.399* | -0.466* | 1.000 |  |  |
| Weight | 0.417* | 0.502* | 0.473* | -0.302* | 1.000 |  |
| Height | 0.309* | 0.338* | 0.404* | -0.475* | 0.560* | 1.000 |
| **Female** |  |  |  |  |  |  |
| Lumbar Spine (LS) | 1.000 |  |  |  |  |  |
| Total Hip  (TH) | 0.737* | 1.000 |  |  |  |  |
| Femoral Neck (FN) | 0.736* | 0.883* | 1.000 |  |  |  |
| Age | -0.556* | -0.555* | -0.612* | 1.000 |  |  |
| Weight | 0.352* | 0.409* | 0.363* | -0.132* | 1.000 |  |
| Height | 0.463* | 0.385* | 0.428* | -0.438* | 0.438* | 1.000 |
