# Supplementary material for: Assessment of peak bone mineral density and its associated factors in Vietnamese adults: A cross-sectional study
Source: PLoS One. 2026 Apr 10;21(4):e0346170. doi: 10.1371/journal.pone.0346170 (PMC13068321; doi:10.1371/journal.pone.0346170)
Supplement: Attachment — Submitted filename: Response letter.docx [file pone.0346170.s004.docx]

March 2, 2025

Editor and reviewers

PLOS ONE

Dear Sir/Madam

On behalf of all authors, we would like to sincerely thank the editors and reviewers for their thoughtful review and valuable suggestions. We greatly appreciate their feedback and have carefully revised the manuscript in accordance with each point raised.

We hope that you now find it suitable for publication in Plos One

Sincerely,

Hoang Thanh Van Nguyen, MPH, Ph.D.
Department of Internal Medicine, University of Medicine and Pharmacy, Hue University,

06 Ngo Quyen Street, Hue City, Thua Thien Hue, Vietnam
Email: [nhtvan@hueuni.edu.vn](mailto:nhtvan@hueuni.edu.vn); nhtvan@huemed-univ.edu.vn

Orcid: 0000-0001-7770-2949

Journal requirements:

When submitting your revision, we need you to address these additional requirements.

1. Please ensure that your manuscript meets PLOS ONE's style requirements, including those for file naming. The PLOS ONE style templates can be found at

<https://journals.plos.org/plosone/s/file?id=wjVg/PLOSOne_formatting_sample_main_body.pdf> and

<https://journals.plos.org/plosone/s/file?id=ba62/PLOSOne_formatting_sample_title_authors_affiliations.pdf>

**Reply 1.** We have gone through the guidelines and revised it thoughtly.

2. We notice that your supplementary tables are included in the manuscript file. Please remove them and upload them with the file type 'Supporting Information'. Please ensure that each Supporting Information file has a legend listed in the manuscript after the references list.

**Reply 2.** We have moved and uploaded as 'Supporting Information”

Reviewer #1: This study systematically established cross-sectional data on the peak bone density (PBD) of adult Vietnamese individuals and the influencing factors, filling the gap in population-specific reference data and having significant value for the prevention and control of osteoporosis. The methods were standardized and the statistics were reasonable. The results revealed key findings such as higher PBD in men than in women and a higher prevalence of lumbar spine disorders.

However, there were limitations:

1.The cross-sectional design makes it difficult to establish causality;

2. The generalizability of the single-center sample is limited;

3.Uncontrolled confounding factors such as dietary calcium intake and physical activity were not controlled. It is recommended to conduct multi-center prospective studies for verification to enhance the national representativeness; references can be made to (Nati. Sci. Rev., 2022, 9, nwac104., ACS nano, 2023, 17(13): 12903-12914.) for strategies in non-amplification, rapid, and highly specific detection, and it is recommended to develop a "real-time detection + AI interpretation" system for osteoporosis risk, shortening the screening cycle.

**Reply 3**: We sincerely thank you for your thoughtful comments and insightful analysis of our manuscript. All the points raised are reasonable, and we have acknowledged them in the limitations section of the discussion. We have also incorporated your suggestions regarding future research directions (page 13, last paragraph).

“Future research should prioritize prospective cohort designs to track longitudinal bone changes and integrate comprehensive lifestyle data to refine predictive models. Building on these models, future initiatives should explore rapid, highly specific detection strategies to develop a 'real-time detection and AI interpretation' system for osteoporosis risk, which could significantly shorten the screening cycle [22,23]. Expanding data collection across diverse geographic regions will be essential to establish a truly representative national reference standard”
